# Comparison of the biomechanical properties of grafts in three anterior cruciate ligament reconstruction techniques based on three-dimensional finite element analysis

**DOI:** 10.1186/s13018-024-04777-x

**Published:** 2024-05-29

**Authors:** Jiawnag Lou, Qi Ma, Xijiu Zhao, Sha Wu, Hong Gao, Wei Zhang, Baojing Zhao, Xu Cai

**Affiliations:** 1 Hebei North University, 11-South Diamond Road, Gaoxin District, Zhangjiakou City, 075000 China; 2 Joint Diseases Center, Beijing Tsinghua Changgung Hospital, School of Clinical Medicine, Tsinghua University, No. 168 Litang Road, Dongxiaokou Town, Changping District, Beijing, 102218 China; 3 Beijing MEDERA Medical Group, Beijing, 102200 China

## Abstract

**Objective:**

To evaluate the biomechanical characteristics of grafts from three different anterior cruciate ligament (ACL) reconstructive surgeries and to determine which method is better at restoring knee joint stability.

**Methods:**

A 31-year-old female volunteer was enrolled in the study. According to the magnetic resonance imaging of her left knee, a three-dimensional model consisting of the distal femur, proximal tibia and fibula, ACL, posterior cruciate ligament, medial collateral ligament and lateral collateral ligament was established. Then, the ACL was removed from the original model to simulate the knee joint after ACL rupture. Based on the knee joint model without the ACL, single-bundle ACL reconstruction, double-bundle ACL reconstruction, and flat-tunnel ACL reconstruction were performed. The cross-sectional diameters of the grafts were equally set as 6 mm in the three groups. The bone tissues had a Young’s modulus of 17 GPa and a Poisson’s ratio of 0.36. The ligaments and grafts had a Young’s modulus of 390 MPa and a Poisson’s ratio of 0.4. Six probes were placed in an ACL or a graft to obtain the values of the equivalent stress, maximum principal stress, and maximum shear stress. After pulling the proximal tibia with a forward force of 134 N, the distance that the tibia moved and the stress distribution in the ACL or the graft, reflected by 30 mechanical values, were measured.

**Results:**

The anterior tibial translation values were similar among the three groups, with the double-bundle ACL reconstruction group performing the best, followed closely by the patellar tendon ACL reconstruction group. In terms of stress distribution, 13 out of 30 mechanical values indicated that the grafts reconstructed by flat bone tunnels had better performance than the grafts in the other groups, while 12 out of 30 showed comparable outcomes, and 5 out of 30 had worse outcomes.

**Conclusion:**

Compared with traditional single-bundle and double-bundle ACL reconstructions, flat-tunnel ACL reconstruction has advantages in terms of stress dispersion. Additionally, flat-tunnel ACL reconstruction falls between traditional double-bundle and single-bundle ACL reconstructions in terms of restoring knee joint stability and is superior to single-bundle ACL reconstruction.

Anterior cruciate ligament (ACL) rupture is a common knee joint injury that occurs during physical activities. In the United States, there are more than 120,000 cases of ACL rupture annually [1]. Currently, the primary treatment for ACL rupture involves surgical ligament reconstruction. Clinically, the main surgical procedures are single-bundle reconstruction and double-bundle reconstruction, which are based on the anatomical understanding that the ACL consists of anterior medial and posterior lateral bundles, which form a double-bundle ligament [2]. However, in recent years, with advancements in anatomical studies of the ACL, some researchers have revealed that the middle part of the ACL is a flat, undivided band-like structure. Its femoral attachment point is elliptical, and the tibial attachment point is either C-shaped or elliptical in structure [3, 4].

Based on current anatomical research, we designed a method for ACL reconstruction using a flat ligament graft and a capsule-shaped bone tunnel, referred to as flat-tunnel ACL reconstruction. To assess the feasibility of this new method and compare it with traditional surgical procedures, we used finite element analysis in this study. We evaluated the stress states of the original knee joints and the knee joints after single-bundle, double-bundle or flat-tunnel ACL reconstruction when the knees were straightly extended and the proximal tibias were anteriorly pulled. Additionally, the distances that the proximal tibias move after pulling were measured and compared between groups, aiming to provide a theoretical basis for further research.

## Materials and methods

### Experimental subjects

A 31-year-old female volunteer was enrolled for the experiment, and three-dimensional thin-slice magnetic resonance imaging (MRI) of her left knee joint was performed (0.4 mm × 0.4 mm × 0.4 mm, Ingenia Elition 3.0 T X, Philips, The Kingdom of the Netherlands). MRI indicated that no structural diseases were present.

### Establishment of the knee joint model

Three-dimensional thin-slice MRI models of the left knee joint of the volunteer were exported as DICOM files. These files were imported into MIMICS (Materialise Company, Belgium) for three-dimensional reconstruction. Three-dimensional models of the femur, tibia, fibula, ACL, posterior cruciate ligament, medial collateral ligament, and lateral collateral ligament were established and then exported in the STL format.

The exported STL files were imported into Geomagic Wrap (Geomagic Corporation, America) to fit the surfaces, generating surface patch models. Features were removed, and the generated smooth surface patches were exported in STP format.

All generated smooth surface patches were imported into SOLIDWORKS (Dassault Systemes Corporation, Franch) for assembly. Overlapping parts between components were removed using Boolean operations, resulting in an original knee joint assembly model. The ACL was removed from the original model to simulate the knee joint after ACL rupture.

**Fig. 1:**
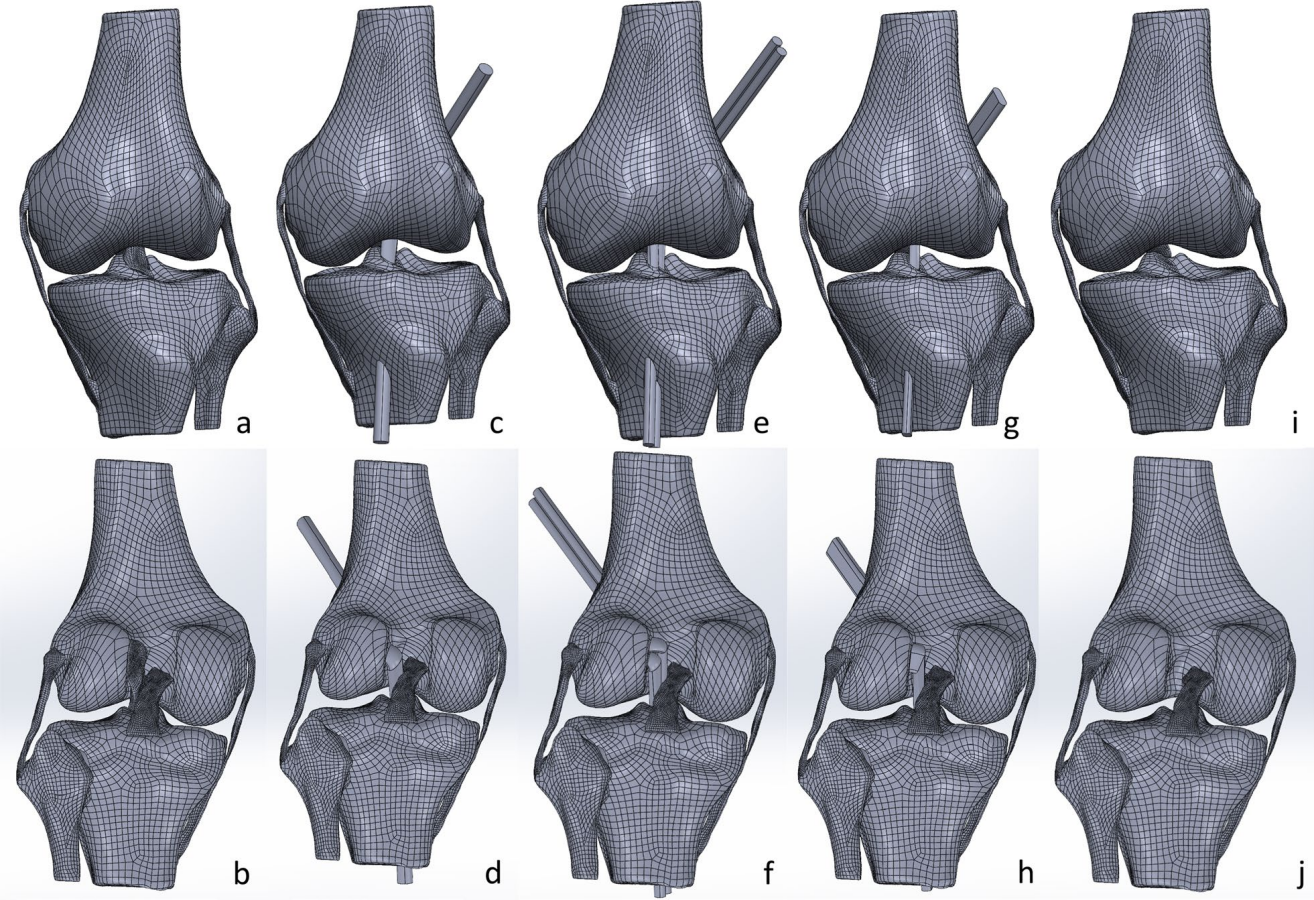
Assembly model diagrams. **a** Original anterior view of the knee joint; **b** posterior view of the original knee joint; **c** anterior view of the single-bundle ACL reconstruction of the knee joint; **d** posterior view of the single-bundle ACL reconstruction of the knee joint; **e** anterior view of the double-bundle ACL reconstruction of the knee joint; **f** posterior view of the double-bundle ACL reconstruction of the knee joint; **g** anterior view of the flat-tunnel ACL reconstruction of the knee joint; **h** anterior view of the flat-tunnel ACL reconstruction of the knee joint; **i** anterior view of the ACL-deficient knee joint; and **j** posterior view of the ACL-deficient knee joint

Single-bundle ACL reconstruction, double-bundle ACL reconstruction, and flat-tunnel ACL reconstruction were performed on the knee joint model without the ACL. The openings of the bone tunnels in the distal femur and proximal tibia were designed according to the femoral and tibial attachment points of the original ACL, and the openings in the proximal femur and distal tibia were positioned on the lateral midpoint of the femoral condyle and on the medial side of the tibial tubercle. The cross-sectional diameter of the graft in single-bundle ACL reconstruction was set as 6 mm. The cross-sectional areas of the grafts in the other two groups were adjusted to be equal to those in the single-bundle group. The graft properties are shown in Table 1. The simulation of the graft passing through the bone tunnel was performed by stretching the tibial stump. To avoid length-related differences in the bone tunnels, all the grafts completely passed through the tunnels. After removing the excess portions inside the joint, the grafts were bent at the femoral attachment point and connected to the tibial attachment point through a lofting operation. The assembled models for each group are shown in Fig. 1. The assembly models were exported as an x_t format file.

**Table 1:** Cross-sectional attributes of transplant vegetation

	Single-bundle ACL reconstruction	Double-bundle ACL reconstruction	Flat-tunnel ACL reconstruction
Cross-sectional area of the tendon graft			
Cross-sectional parameters (mm)	R_1_ = 6.000 0	R_2_ = R_3_ = 4.242 6	a = 3.475 4 b = 5.406 1
Cross-sectional area (mm^2^)	28.274 3	28.239 1	28.338 2

### Establishment and configuration of finite element models

The previously generated x_t format file was imported into the finite element analysis software Ansys (Ansys Corporation, America). A linear elastic model was used for analysis, and the material properties of the different components were determined based on a previously published study by Achilles Vairis et al. [5]; that is, the bone material had a Young’s modulus of 17 GPa and a Poisson’s ratio of 0.36, and the ligaments and graft materials had a Young’s modulus of 390 MPa and a Poisson’s ratio of 0.4.

The contact conditions were defined as follows: the original ACL attachment points were bound to the femur and tibia; the grafts within the bone tunnels were bound to the femur and tibia; the attachment points of the medial collateral ligament and posterior cruciate ligament were bound to the femur and tibia; and the attachment points of the lateral collateral ligament were bound to the femur and fibula. The medial collateral ligament, except for its attachment points, had frictionless contact with the tibia. The proximal 1/3 of the femur was set to be absolutely fixed. The connection between the tibia and the fibula was set to be relatively fixed.

After adjusting the mesh size of the model, we set the mesh element size to 2 mm for bone tissues and 1 mm for ligaments and grafts.

### Loading and solution

Because the primary function of the ACL is to limit anterior translation of the tibia, and clinically, ACL rupture is often assessed by fixing the thigh and anteriorly pulling the tibia, a forward force of 134 N was applied to the proximal tibia to identify differences between groups.

Model solutions were generated in ANSYS. For the original ACL, the probes were placed at the posterosuperior and anteroinferior parts of the femoral attachment point, at the anteromedial and posterolateral parts of the middle ligament, and at the anteromedial and posterolateral parts of the tibial attachment point (a total of six probes). For the grafts, the probes were placed at the posterosuperior and anteroinferior parts of the distal opening of the femoral bone tunnel, at the anteromedial and posterolateral parts of the middle graft, and at the anteromedial and posterolateral parts of the proximal opening of the tibial bone tunnel (a total of six probes). These probes were used to measure the force distribution in ligaments and grafts and to obtain the values of equivalent stress, maximum principal stress, and maximum shear stress. The distance the tibia moved anteriorly after being pulled was measured five times to assess the constraint effect of the graft on the tibia.

## Results

**Fig. 2:**
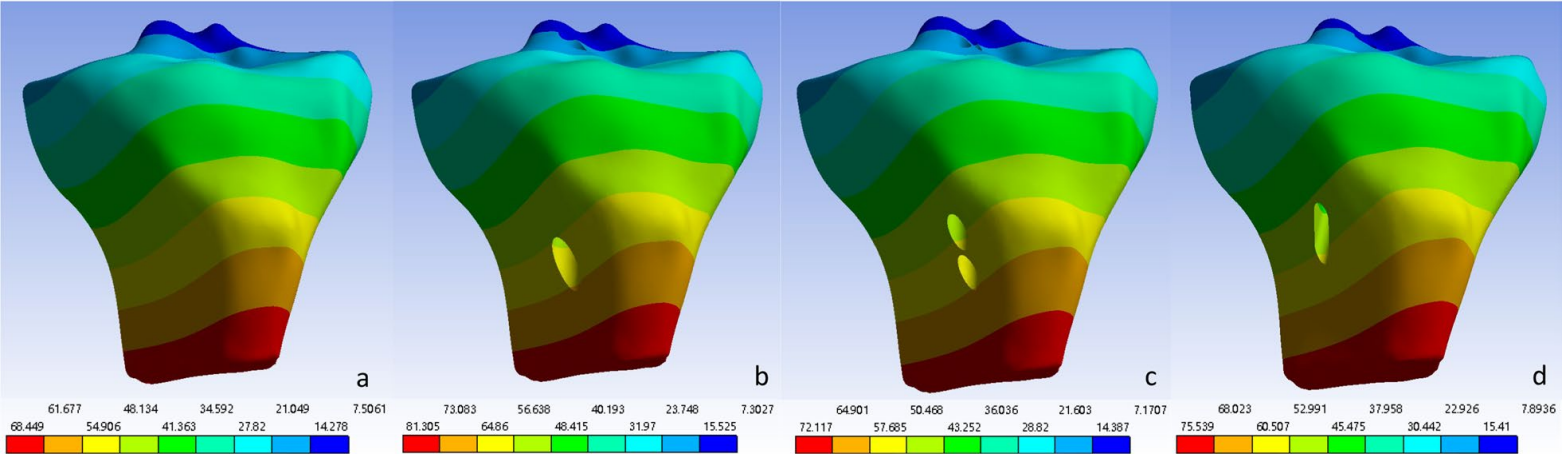
Tibial displacement of each group. **a** Original knee joint tibial displacement cloud map; **b** Single-bundle ACL reconstruction knee joint tibial displacement cloud map; **c** Double-bundle ACL knee joint tibial displacement cloud map; **d** Flat-tunnel ACL reconstruction knee joint tibial displacement cloud map

The anterior tibial movement of each model determined via Ansys software is shown in Fig. 2 and Table 2. The average anterior tibial translation in the original knee joint was 35.4420 mm. After single-bundle ACL reconstruction, it was 41.2630 mm long. After double-bundle ACL reconstruction, the length was 37.0360 mm. After flat-tunnel ACL reconstruction, it was 39.0170 mm long. The analysis could not be performed on the knee joint model without the ACL, which was likely due to model damage during the solution process. Therefore, it could be concluded that among the three ACL reconstruction methods mentioned, there was no significant difference in restoring knee joint stability, with double-bundle ACL reconstruction being the best and flat-tunnel ACL reconstruction being closest to it.

**Table 2:** Anterior translation values of each group of tibia (mm)

	Native knee joint	Single-bundle ACL reconstruction	Double-bundle ACL reconstruction	Flat-tunnel ACL reconstruction	ACL-deficient knee
Max	68.449	79.75	72.117	75.539	Unable to solve
Min	7.506 1	8.449 8	7.170 7	7.893 6	
Mean	35.442	41.263	37.036	39.017	

**Fig. 3:**
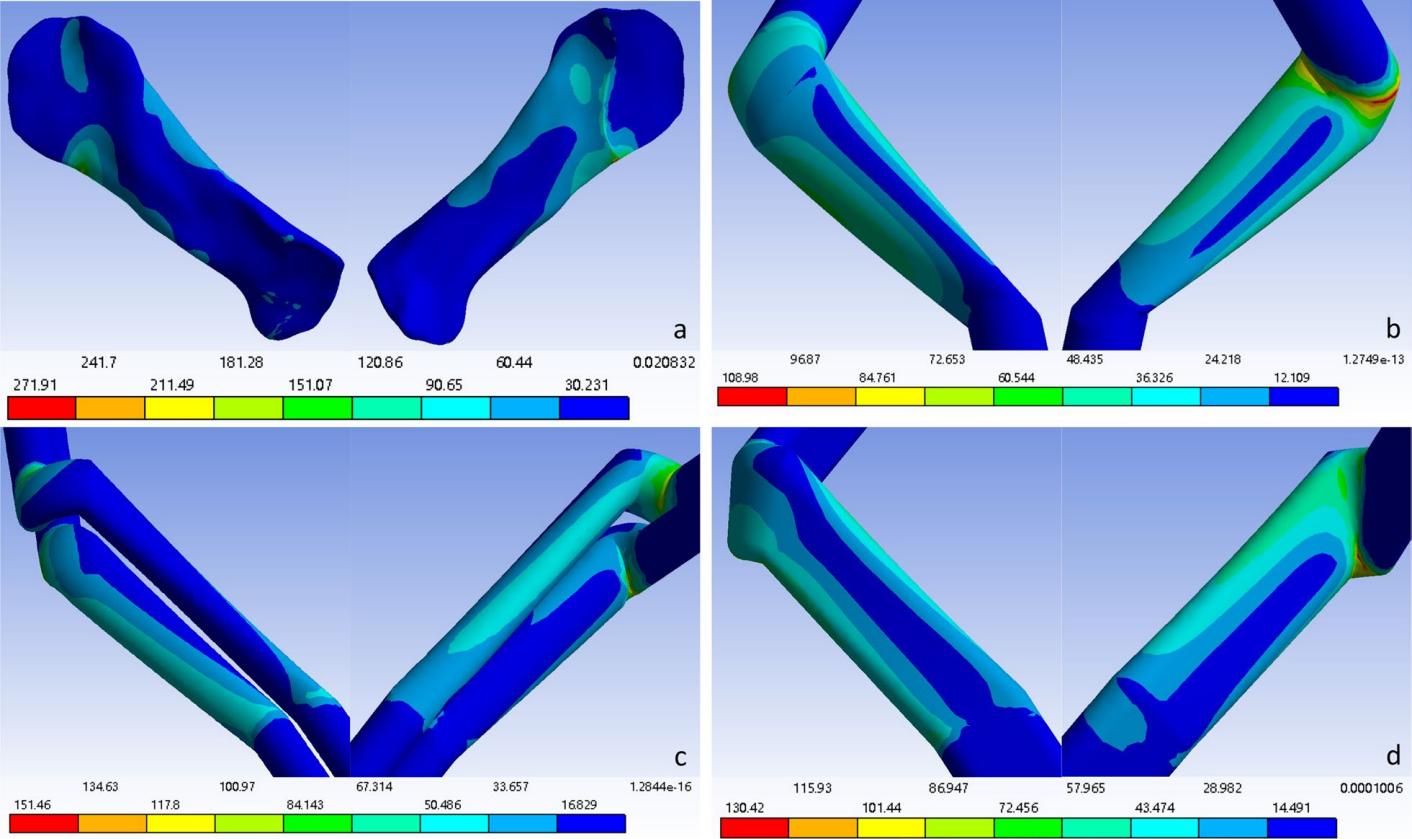
Distribution cloud map of the original ACL/graft equivalent stress. **a** Original ACL stress cloud map; **b** Single-bundle ACL reconstruction graft stress cloud map; **c** Double-bundle ACL reconstruction graft stress cloud map; **d** Flat-tunnel ACL reconstruction graft stress cloud map

**Fig. 4:**
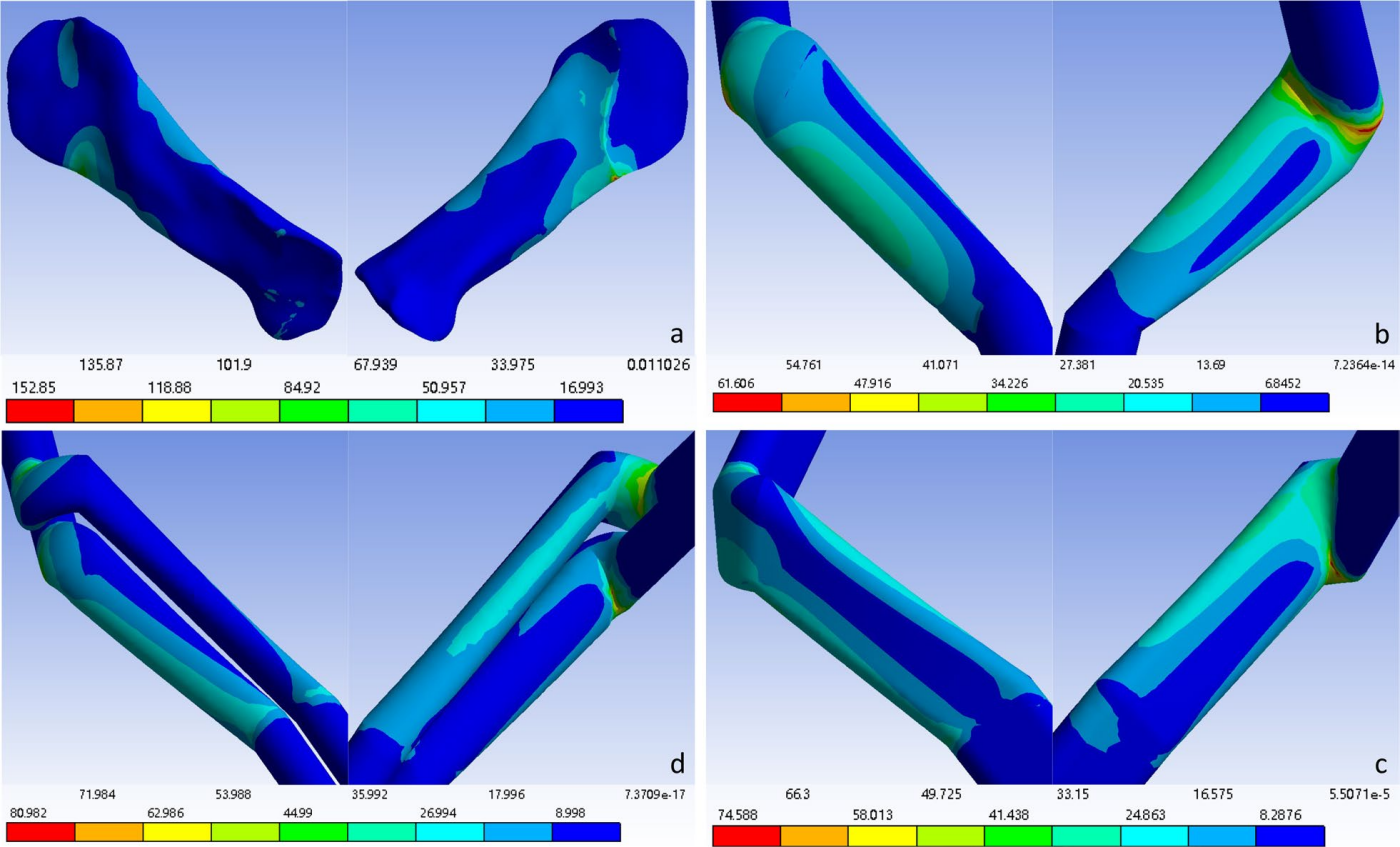
Distribution cloud map of the maximum principal stress of the ACL/graft. **a** Original ACL stress cloud map; **b** Single-bundle ACL reconstruction graft stress cloud map; **c** Double-bundle ACL reconstruction graft stress cloud map; **d** Flat-tunnel ACL reconstruction graft stress cloud map

**Fig. 5:**
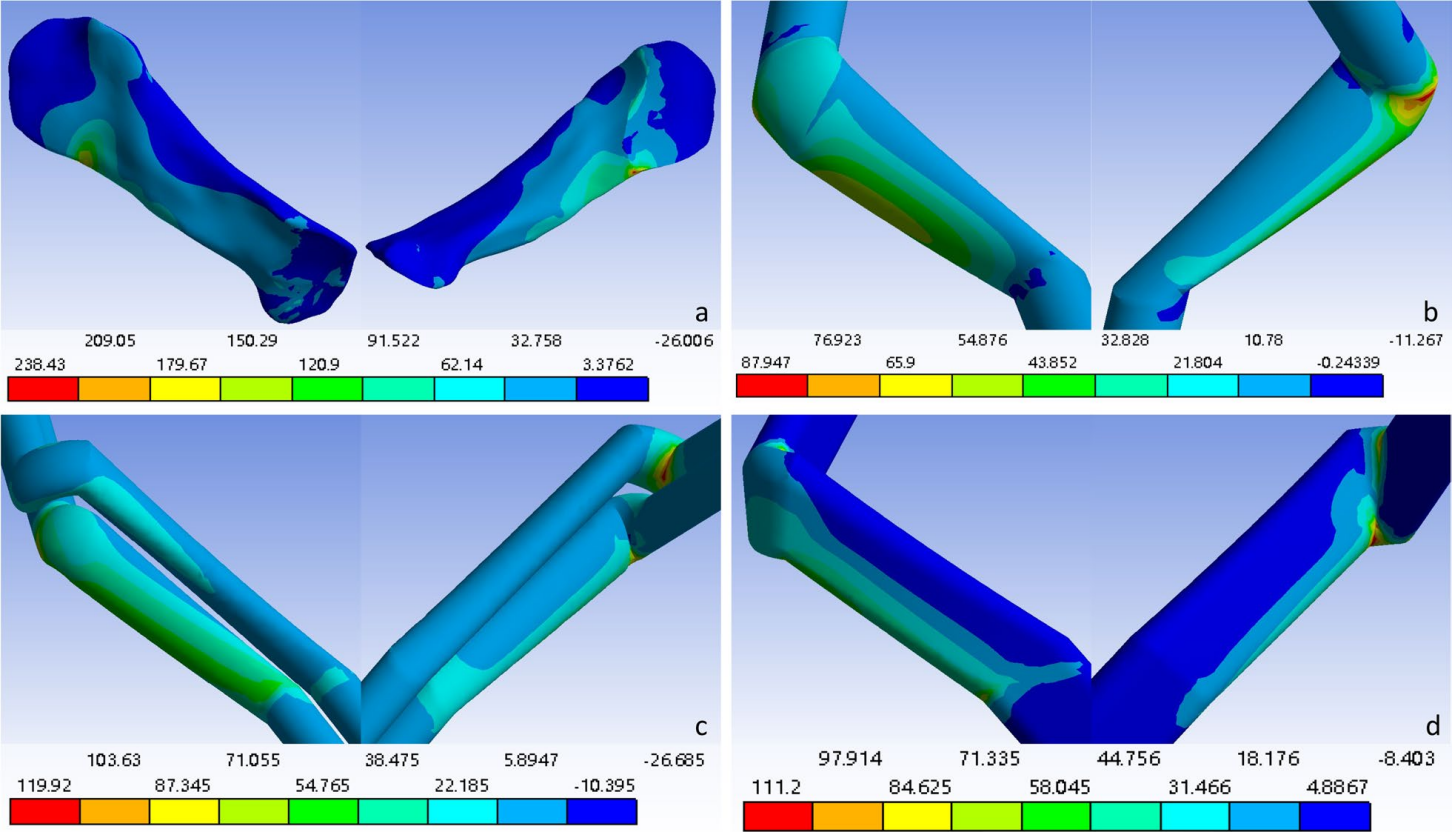
Distribution cloud map of the maximum shear stress of the ACL/graft. **a** Original ACL stress cloud map; **b** Single-bundle ACL reconstruction graft stress cloud map; **c** Double-bundle ACL reconstruction graft stress cloud map; **d** Flat-tunnel ACL reconstruction graft stress cloud map

The force distributions at the detection points of the ligaments and grafts in each model after solution treatment are shown in Table 3. Since greater stress on ligaments or grafts is more likely to lead to rupture, a lower force corresponds to a lower risk of rupture. At the posterosuperior part of the original ACL or at the distal opening of the femoral bone tunnel after reconstruction, the flat-tunnel ACL reconstruction model had a better maximum principal stress than did the other three groups, while the other stress levels were comparable to those of the other groups. At the anteroinferior part of the original ACL or at the distal opening of the femoral bone tunnel, the flat-tunnel ACL reconstruction model had better levels of maximum principal stress and cross-sectional maximum shear stress than did the other three groups, while other stress levels were comparable among the groups. At the anteromedial part of the middle original ACL or the middle graft after reconstruction, the flat-tunnel ACL reconstruction model had better equivalent stress and coronal maximum shear stress than did the other three groups, but the cross-sectional and sagittal maximum shear stress were worse in the flat-tunnel ACL reconstruction model than in the other groups, and other stress levels were comparable among the groups. At the posterolateral part of the middle original ACL or the middle graft after reconstruction, the flat-tunnel ACL model had better stress levels than did the other three groups. At the anteromedial part of the original ACL or the proximal opening of the tibial bone tunnel, the flat-tunnel ACL model had a better level of sagittal maximum shear stress than did the other three groups, while the equivalent stress and coronal maximum shear stress were worse in the flat-tunnel ACL model than in the other groups, and other stress levels were comparable among the groups. At the posterolateral part of the original ACL or the proximal opening of the tibial bone tunnel, the flat-tunnel ACL model had a better level of cross-sectional maximum shear stress than did the other three groups, but the level of coronal maximum shear stress was worse in the flat-tunnel ACL model than in the other three groups, and other stress levels were comparable among the groups. In summary, 30 stress measurements at 6 detection points were performed in one ligament or graft, among which 13 measurements indicated that the graft in the flat-tunnel ACL reconstruction model was superior, 12 measurements fell between those in the other three groups, and 5 measurements presented worse outcomes than those in the other three groups. The stress maps of the equivalent stress, maximum principal stress, and maximum shear stress of the graft are shown in Figs. 3, 4, and 5, respectively.

**Table 3:** Stress values at each detection point of each group

		Femoral ligament insertion /tunnel entrance		Ligament/transplant tendon midsection		Tibia ligament insertion /tunnel entrance	
		Posterior superior	Anterior inferior	Anterior medial	Posterior superior	Anterior inferior	Anterior medial
Von mises stress	Native knee joint	46.5420	271.9100	57.4960	37.4070	0.5119	19.2310
	Single-bundle ACL reconstruction	33.2690	108.9800	28.0650	30.3350	4.6063	15.5380
	Double-bundle ACL reconstruction	85.3690	67.2180	28.9180	12.5810	9.8051	39.4490
	Flat-tunnel ACL reconstruction	40.9380	89.2240	15.5910	10.1100	14.4810	21.3340
Maximum principal stress	Native knee joint	68.8720	238.4300	0.2119	36.8500	0.5694	16.0130
	Single-bundle ACL reconstruction	19.0240	85.9510	0.2978	30.3390	4.7469	1.0786
	Double-bundle ACL reconstruction	31.8360	54.1320	0.0074	12.5810	0.5580	29.6220
	Flat-tunnel ACL reconstruction	17.2390	36.9770	0.0573	9.9821	2.0152	19.8050
Maximum sagittal plane shear stress	Native knee joint	0.0769	− 141.3000	− 0.1525	− 1.2799	0.2753	− 9.0907
	Single-bundle ACL reconstruction	− 7.2159	− 34.0120	− 0.3412	− 1.0924	0.6065	0.7618
	Double-bundle ACL reconstruction	26.8500	− 14.3360	0.2231	− 0.3901	0.5164	− 8.2421
	Flat-tunnel ACL reconstruction	12.2110	− 48.5630	− 0.7386	− 0.1159	− 0.1801	0.8634
Maximum coronal plane shear stress	Native knee joint	0.2283	− 46.8630	21.7650	− 16.2480	− 0.0747	9.6595
	Single-bundle ACL reconstruction	4.6391	− 14.2120	13.8440	− 14.4480	0.5629	4.5813
	Double-bundle ACL reconstruction	0.2456	− 14.1070	13.3760	− 6.1397	3.5937	− 9.6595
	Flat-tunnel ACL reconstruction	2.9689	2.4550	6.5190	− 5.0477	5.2811	− 10.2620
Maximum transverse plane shear stress	Native knee joint	4.1823	− 46.5280	− 0.0577	− 1.9529	− 0.0359	− 2.9897
	Single-bundle ACL reconstruction	− 9.5700	− 44.6750	− 0.1365	− 0.7162	2.2012	− 5.2783
	Double-bundle ACL reconstruction	33.6460	-19.0830	0.2974	-0.4532	1.1221	5.3182
	Flat-tunnel ACL reconstruction	9.0128	1.8030	− 1.2672	− 0.3249	− 0.9944	2.2017

The three stress peaks in the original ACL ligament or the graft appeared at the anteroinferior part of the femoral attachment point or at the distal opening of the femoral bone tunnel, consistent with Kazunori Yasud et al.’s study, which indicated that greater stress appeared on the posterolateral bundle of the ACL in a straightly extended knee joint [6].

## Discussion

Anatomical reconstruction of the ACL was initially reported by Konsei Shino et al. in 2005, utilizing a bone–patellar tendon–bone graft and rectangular bone tunnel for ACL reconstruction [7]. Subsequently, this technique was also applied by the same authors in revision surgery following failed single-bundle ACL reconstruction, as described in subsequent reports [8]. In 2015, Domnick et al. confirmed that flattening the circular tendon does not affect its structural characteristics [9]. Subsequently, researchers began using semitendinosus tendon grafts for anatomical ACL reconstruction, constructing a special C-shaped tibial tunnel and an elliptical femoral tunnel and achieving favourable outcomes [10]. The flat-tunnel ACL reconstruction model constructed in this study is a simplification based on this method combined with traditional ACL reconstruction techniques, aiming for a straightforward approach to maximize the restoration of knee joint function and stability.

This study used finite element analysis to compare the functionality of the original knee joint, two traditional ACL reconstructions (single-bundle and double-bundle), and the knee joint after flat-tunnel ACL reconstruction, as well as the comprehensive force distribution on ligaments and grafts. From this study, it is evident that in this finite element analysis model, the stress distribution of the graft in flat-tunnel ACL reconstruction is generally superior to that of the two traditional ACL reconstructions. Additionally, in terms of restoring knee joint stability, flat-tunnel ACL reconstruction falls between the two traditional ACL reconstructions.

Considering the stress distribution maps and stress data, we believe that the observed stress differences in this experiment are related to the cross-sectional shape of the graft. Compared to the circular cross section of the double-bundle ACL graft, the capsule-shaped cross section of the flat-tunnel ACL graft is more conducive to stress conduction, dispersing stress and reducing concentration. This can decrease the risk of graft rupture postoperatively. Research by Rafał Trąbka et al. suggested that double-bundle ACL reconstruction can better restore knee joint stability than can single-bundle reconstruction but may be inferior in terms of functionality [11]. This finding aligns with the conclusion drawn in this study that under the condition of equal graft cross-sectional area, flat-tunnel ACL reconstruction is close to double-bundle ACL reconstruction and superior to single-bundle ACL reconstruction in restoring knee joint stability.

In clinical practice, double-bundle ACL reconstruction requires drilling two sets of adjacent tunnels, while single-bundle ACL reconstruction and flat-tunnel ACL reconstruction require drilling one set of tunnels. Therefore, compared to the latter, double-bundle ACL reconstruction is more challenging in terms of surgical techniques, requiring precise planning of tunnel locations, leading to a greater failure rate and a greater risk of damage to bony tunnels. In contrast, single-bundle ACL reconstruction is simpler and has a lower failure rate but performs poorly in restoring knee joint function. Flat-tunnel ACL reconstruction combines the advantages of both methods, aiming to maximize knee joint stability through the simplest surgical procedure. Moreover, the graft used for flat-tunnel ACL reconstruction closely resembles the original ACL structure, allowing anatomical reconstruction.

This study has limitations, including the use of reconstructed structures such as the distal femur, proximal tibia, proximal fibula, medial collateral ligament, lateral collateral ligament, ACL, and posterior cruciate ligament. Due to the limited scanning range of MRI, the model established in this study cannot cover all functional structures around the knee joint. It can only partially reflect the real situation of the knee joint and cannot achieve complete restoration. In future research studies, animal models will be employed. The participants who were recruited for this study were Han Chinese females with average development. Therefore, the knee joint model constructed in this study has a certain representativeness among Han Chinese females. However, for males and non-Han ethnic groups, further research is needed to validate the data generated in this study. Additionally, the inability to analyse the ACL-deficient knee joint in the software, which was considered to be due to model damage during calculation, results in a lack of a negative control group in the study. Improvements in analytic approaches to obtain negative control group data will be addressed in future research.

## Conclusion

From this study, it can be concluded that flat-tunnel ACL reconstruction has advantages in terms of stress dispersion compared to traditional single-bundle and double-bundle ACL reconstructions. This approach may reduce the risk of graft rupture postoperatively. Additionally, flat-tunnel ACL reconstruction falls between traditional double-bundle and single-bundle ACL reconstructions in terms of restoring knee joint stability and is superior to single-bundle ACL reconstruction.

## Data Availability

All the data generated or analysed during this study are included in this published the article.
